# Reoperative Aortic Arch Surgery under Mild Systemic Hypothermia: Two-Center Experience

**DOI:** 10.1055/s-0041-1725073

**Published:** 2021-10-07

**Authors:** Petar Risteski, Isabel Radacki, Andreas Zierer, Aris Lenos, Anton Moritz, Paul P. Urbanski

**Affiliations:** 1 Department of Thoracic and Cardiovascular Surgery, Johann Wolfgang Goethe University, Frankfurt am Main, Germany; 2 Department of Cardiac, Vascular, and Thoracic Surgery, Kepler University Hospital, Linz, Austria; 3 Department of Cardiovascular Surgery, Cardiovascular Clinic Bad Neustadt, Bad Neustadt, Germany

**Keywords:** aortic arch surgery, reoperation, mild hypothermia

## Abstract

**Background** The aim of the study was to assess the indications, surgical strategies, and outcomes after reoperative aortic arch surgery performed generally under mild hypothermia.

**Methods** Ninety consecutive patients (60 males, mean age, 55 ± 16 years) underwent open reoperative aortic arch surgery after previous cardiac aortic surgery. The indications included chronic-progressive arch aneurysm (55.5%), chronic aortic dissection (17.8%), contained arch rupture (16.7%), and graft infection (10%). The reoperation was performed through a repeat sternotomy (96%) or clamshell thoracotomy (4%) using antegrade cerebral perfusion under mild systemic hypothermia (28.9 ± 2.5°C) in all except three patients.

**Results** The surgery comprised hemiarch or total arch replacement in 41 (46%) and 49 (54%) patients, respectively. The distal extension included classic or frozen elephant trunk technique, each in 12 patients, and total descending aorta replacement in 4 patients. Operative mortality was 6 (6.7%) among all patients, with age identified as the only independent predictor of operative mortality ( *p* = 0.05). Permanent and transient neurologic deficits occurred in 1% and 9% of the patients, respectively. Estimated survival at 8 years was 59 ± 8% with advanced heart failure predictive for late mortality ( *p* = 0.014). Freedom from second reoperation or intervention on the aorta was 78 ± 6% at 8 years, with most of these events occurring downstream in patients with chronic degenerative aneurysms.

**Conclusion** Aortic arch reoperations performed using antegrade cerebral perfusion under mild systemic hypothermia offer favorable operative outcomes with an exceptionally low rate of neurologic morbidity without any difference between hemiarch and complex arch procedures.

## Introduction

Reoperative aortic arch surgery (RAAS) after prior cardiac aortic surgery is considered a technically challenging and high-risk procedure for many reasons, due to the danger associated with the repeat sternotomy, an adhesions-related hostile operative field and complex pathology extending to the descending aorta. 1 2 3 4 5 6 7 8 9 Lastly, there is a perceived need for prolonged circulatory arrest for which deep 1 2 3 4 or moderate hypothermia 5 6 7 8 9 is usually used for the brain and organ protection. Some of the well-recognized drawbacks of deep systemic hypothermia include longer cardiopulmonary bypass (CPB) times, severe coagulopathy requiring transfusions, reexplorations for bleeding, and longer intensive care and hospital stays. 9 10 11

Several recent reports on reoperative aortic surgery showed that prolong CPB time is an independent predictor of operative 6 8 and late mortality. 12 Mild systemic hypothermia shortens the CPB times needed for cooling and rewarming of patient, and may favorably influence early outcomes. To date, however, there are no reports on RAAS in mild systemic hypothermia.

The aim of this study was to assess the effectiveness of this protection strategy in RAAS. Furthermore, we investigate the indications and timing for this type of surgery, identify risk factors for early and late mortality, as well as for second reoperation, and evaluate the early and late outcomes in a cohort of patients reoperated on the aortic arch in mild systemic hypothermia.

## Materials and Methods

The study cohort of this investigation comprises 90 patients who underwent RAAS after surgery on the proximal aorta (defined as replacement of the ascending aorta with open distal anastomosis or any form of open aortic arch repair) using antegrade cerebral perfusion (ACP) with mild systemic hypothermia (28–30°C) in two aortic referral centers in Germany, Bad Neustadt (observation period between 2002 and 2018) and Frankfurt am Main (observation period between 2000 and 2018).

We excluded patients who had previously undergone nonaortic operations like coronary bypass grafting on mitral or aortic valve procedures. The indications and type of the primary operation are shown in Table 1 . The study was approved by the ethics committees of both institutions and individual consent was obtained. For purposes of this study, we used the hospital electronic medical files of the patients and our aortic surgery database.

**Table 1:** Indications and type of primary operation in patients undergoing reoperative aortic arch surgery

Characteristics	*n*	(%)
*Indications for the primary operation* :		
Acute Type A aortic dissection	44	48.9
Degenerative aortic aneurysm	38	42.2
Aortic coarctation	8	8.9
*Type of primary operation* :		
RAA	23	25.6
AVR + RAA	34	37.8
Aortic valve reconstruction + RAA	5	5.6
David procedure + RAA	1	2.7
Bentall procedure + RAA	2	5.6
RAA + hemiarch replacement	8	22.2
AVR + RAA + hemiarch replacement	1	2.7
Hemiarch replacement	2	5.6
Hemiarch replacement + David procedure	1	2.7
RAA + total arch replacement	5	13.9
Distal arch replacement for correction of aortic coarctation	8	8.9

Abbreviations: AVR, aortic valve replacement; RAA, replacement of the ascending aorta.

There were 60 (67%) men and 30 (33%) women, with a mean age of 55 ± 16 years. The patients' demographic data are shown in Table 2 . All patients underwent preoperative computed tomography (CT) to delineate the extent of the aortic pathology and plan reentry in thorax, in addition to transthoracic echocardiography which was used to assess cardiac and valve function. Furthermore, coronary angiography was performed in all elective cases, and, if feasible, in some urgent cases.

**Table 2:** Demographic characteristics of the study patients

Characteristics	*n*	(%)
NYHA classes III and IV	24	26.7
COPD (GOLD classes II–IV)	13	14.4
Systemic hypertension	83	92.2
Compensated chronic renal insufficiency	16	17.8
Decompensated chronic renal insufficiency	3	3.3
Chronic peripheral vascular disease	5	5.6
Extracranial carotid disease (>50% luminal stenosis)	4	4.4
Diabetes mellitus	9	10
Preoperative neurologic dysfunction	5	5.6
*Etiology* :		
Marfan's syndrome	5	5.6
Degeneration	70	77.8
Aortitis	4	4.4
Graft infection	9	10
Iatrogenic	2	2.2
*Indication for reoperation* :		
Degenerative aortic arch aneurysms	50	55.5
Contained rupture of aortic arch	15	16.7
Chronic postdissection aortic arch aneurysm	16	17.8
Graft infection	9	10
*Aortic valve* :		
Aortic valve stenosis	6	6.6
Aortic valve insufficiency	36	40

Abbreviations: COPD, chronic obstructive pulmonary disease; GOLD, global initiative for chronic obstructive lung disease; NYHA, New York Heart Association.

### Perioperative Management

The standardized surgical, perfusion, temperature, and monitoring protocols used in both institutions have been previously described in detail. 13 14 15 Both institutions used cannulation of a supra-aortic branch for arterial return and uninterrupted ACP, as well as mild systemic hypothermia with target core temperature of 28 to 30°C. Standard methods for resternotomy have been used. 16

In Frankfurt, the right axillary artery was considered the primary cannulation site. The artery was exposed in the deltopectoral groove and directly cannulated with an 18- 22-F flexible arterial cannula (Edwards Lifesciences, Irvine, CA). Following chest reentry, the right atrium was cannulated in a standard fashion. CPB was started, and cooling was limited to 28 to 30°C rectal or bladder temperature. After diastolic cardiac arrest with cold blood cardioplegia given in both antegrade and retrograde fashion, the aortic repair was commenced. The innominate and left carotid arteries were freed from adhesion and snared with silicone elastomer loops and occluded at the initiation of selective ACP. After opening the aortic arch, the left subclavian artery was blocked with an adequately sized Fogarty catheter to prevent cerebral steal and obtain a bloodless operative field. In patients with bilateral antegrade cerebral perfusion, a balloon-tipped flexible cannula connected to the arterial CPB line was placed into the left common carotid artery for additional perfusion of the left hemisphere. Selective ACP was performed with a perfusate temperature of 28 to 30°C in a pressure-controlled manner. The perfusion pressure was kept above 75 mm Hg, allowing for a flow of 1.3 ± 0.3 L/min. At that point, the aortic arch procedure was performed. The preferred technique of dealing with the supra-aortic vessels was en bloc reimplantation as the Carrel patch. For distal aortic repair, conventional or frozen elephant trunk using the E-Vita Open Plus prosthesis (Jotec GmbH, Hechingen, Germany) was applied where appropriate. Once aortic arch repair was completed, the arch prosthesis was carefully deaired and reconstitution of full body perfusion was initiated.

In Bad Neustadt, cannulation of the common carotid artery through a separate neck incision was used for arterial return for CPB. The innominate artery was considered an alternative cannulation site. Following heparin administration, the exposed segment of the artery was cross-clamped and an 8- or 10-mm polyester graft was anastomosed to the artery. Distal body circulatory arrest during selective ACP was used for arch replacement. The deepest temperature was determined by the surgeon, in accordance with the expected period of circulatory arrest, mainly aiming at 28 to 30°C rectal temperature. The ACP was always performed with an arterial blood temperature of 28°C. Unilateral antegrade cerebral perfusion (UACP) was set at a perfusion pressure on the pump unit of 80 mm Hg, allowing for a UACP flow of 1.2 ± 0.2 L/min when the left common carotid artery was used for perfusion and, considering the flow to the right arm, a flow of 1.7 ± 0.3 L/min when the innominate artery or the right common carotid artery was used. During rewarming, the **Y** -shaped arterial line was used to switch the arterial perfusion from the cannulated artery to the aortic graft. Isolated anastomosing of supra-aortic vessels is the preferred technique for total arch replacement in Bad Neustadt for which vascular prosthesis with four-side branches (InterGard AorticArch; InterVascular, La Ciotat, France) is used.

In both institutions, the temperature gradient between the oxygenated blood (arterial line) and the patient's core temperature during rewarming was set at a maximum of 10°C, with a peak temperature of blood leaving the oxygenator of 38.5°C. The acid–base balance was maintained using the α-stat method throughout the operation. Neurovascular monitoring varied throughout the study period and consisted of frontal cerebral saturation assessment using near-infrared spectroscopy in the latter half of the series, as well as pressure measurement, in at least one radial artery. Concomitant cardiac procedures were performed during cooling and rewarming. Four senior surgeons performed all procedures.

### Definitions

Surgery was defined as emergency if the patient was transferred immediately to the operating room once the diagnosis was made, and urgent when performed within 24 hours after diagnosis. All other cases were considered elective. Operative mortality was defined as death within 30 days after the operation, or at any time during the observed hospitalization. Temporary neurologic dysfunction (TND) was defined as the presence of reversible postoperative motor deficit, confusion, agitation, or transient delirium. The CT findings were required to be normal, with resolution of all symptoms before discharge. Permanent neurologic deficit (PND) was defined as the presence of either new focal (stroke) or global (coma) permanent neurologic dysfunction. Mechanical ventilation was considered prolonged if the patient was ventilated longer than 72 hours.

### Follow-up and Statistical Analysis

Survivors were followed up in the outpatient clinics of both units or by contacting the referring cardiologist or general practitioner. A CT scan was performed annually. All statistical calculations have been performed with the SPSS Version 22.0 (SPSS, Chicago, IL). Categorical variables are expressed as percentages and continuous variables are expressed as mean ± standard deviation throughout this paper. Statistical significance was calculated using the Wilcoxon Mann–Whitney test and defined for ≤0.05. Univariate and multivariate logistic regression analyses have been used to identify risk factors predictive of operative and late mortality, as well as late aortic event like second reoperation or intervention. Survival and freedom from second reoperation or intervention on the aorta were calculated using the standard Kaplan–Meier analysis.

## Results

The elapsed time between the primary operation and the reoperation averaged 9 ± 8 years in this population of patients, and was nonsignificantly shorter ( *p* = 0.46) in patients with degenerative aortic aneurysm (6 ± 6 years) when compared with patients with Type A aortic dissection (9 ± 6 years). The reoperation was conducted either on elective (49 patients, 54%), urgent (33 patients, 37%) or emergent basis (8 patients, 9%).

### Operative Results

Hemiarch replacement was performed in 41 (46%) patients. The remaining 49 (54%) patients received total arch replacement, further extended with either conventional in 12 (13%), or frozen elephant trunk procedure in 12 (13%) patients, or conventional replacement of the descending aorta in 4 (4%) patients. Associated procedures are listed in Table 3 . The cardiopulmonary bypass, aortic cross clamp and ACP times averaged 228 ± 74, 123 ± 53, and 48 ± 29 minutes, respectively. Unilateral ACP was applied in the majority of the patients (80, 89%). The mean lowest systemic temperature was 28.9 ± 2.5°C.

**Table 3:** Intraoperative details

Characteristics	Mean ± SD	
Operative time (min)	455 ± 115	
Cardiopulmonary bypass time (min)	228 ± 74	
Aortic cross clamp time (min)	123 ± 53	
Antegrade cerebral perfusion time (min)	48 ± 29	
Lowest systemic temperature (°C)	28.9 ± 2.5	
*Arterial cannulation site* :	*n*	(%)
Common carotid artery	45	50
Right axillary artery	32	32.5
Common carotid and femoral artery	7	7.8
Femoral artery	7	4.4
Brachiocephalic trunk	2	2.2
Hemiarch replacement	41	45.5
*Total arch replacement* :	*n*	(%)
Total arch replacement and conventional elephant trunk	49	54.4
Total arch replacement and frozen elephant trunk	12	13.3
Total arch and conventional descending aorta replacement	4	4.4
*Associated procedures* :	*n*	(%)
David procedure	3	3.3
Bentall procedure	22	24.4
Aortic valve repair	8	8.9
Aortic valve replacement	22	24.4

Abbreviation: SD, standard deviation.

Reexploration for bleeding was required in 12 (13%) patients. The most common postoperative morbidity was renal failure in 19 (21%) patients, necessitating temporary or permanent dialysis in 15 (17%) and 4 (4%) patients, respectively, followed by prolonged mechanical ventilation in 15 (17%) patients ( Table 4 ). New TND was observed in eight (9% patients) and new PND was detected in one (1%) patient with hemiplegia. Spinal cord injury did not occur in this population of patients.

**Table 4:** Postoperative outcomes

Characteristics	*n*	%
*Operative mortality* :	6	6.7
Cardial	4	4.4
Multiorgan failure	2	2.2
Reexploration for bleeding	12	13.3
Prolonged mechanical ventilation	15	16.7
New malperfusion syndrome	2	2.2
*Acute renal failure with dialysis* :		
Temporary	15	16.7
Permanent	4	4.4
New temporary neurologic deficit	8	8.9
*New permanent neurologic deficit* :		
Hemiplegia	1	1
Spinal cord injury	0	0
Laryngeal nerve palsy	8	8.9
Phrenic palsy	1	1
Postoperative Intensive care unit stay (d)	10 ± 12	
Postoperative hospital stay (d)	22 ± 14	

Operative mortality was observed in six (6.7%) patients, due to heart failure in four (4%) and multiple organ failure in two (2%) patients. Univariate analysis identified age ( *p* = 0.027) and chronic obstructive pulmonary disease (COPD; *p* = 0.035, odds ratio [OR] = 0.127, 95% confidence interval [95% CI]: 0.019–8.667) as significant predictors of early mortality, while CPB time approached significance ( *p* = 0.08). In the multivariate analysis, age ( *p* = 0.05, OR = 0.889, 95% CI: 0.79–1) independently predicted operative mortality, while CPB time ( *p* = 0.065, OR = 0.988, 95% CI: 0.976–1.001), and COPD ( *p* = 0.095, OR = 6.576, 95% CI: 0.72–60.14) approached significance.

### Late Outcomes

Eighty-four survivors were followed for 364 patient-years. Mean follow-up for survivors was 52 ± 43 (range: 2–198) months.

**Fig. 1:**
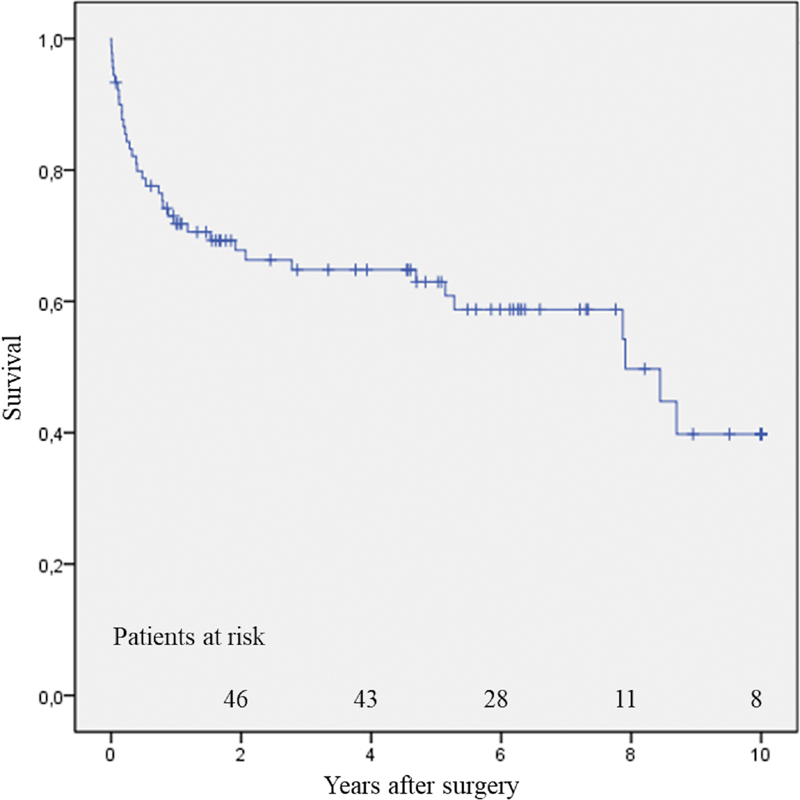
Late survival.

Twenty-eight patients died during follow-up, due to sepsis in five, heart failure in five, cancer in four, pneumonia in two, aortic rupture in two, stroke in two, mesenteric infarction in one, trauma in one, and multiorgan failure in one. In five patients, the cause of death remained unknown. Survival was 79 ± 4% at 1 year, 74 ± 5% at 4 years, and 59 ± 8% at 8 years ( Fig. 1 ). Univariate analysis identified chronic heart failure with advanced functional impairment (defined as New York Heart Association (NYHA) classes III and IV; *p* = 0.037, OR = 2.944, 95% CI: 1.066–8.135) as a significant predictor of late mortality, while chronic postdissection aneurysm ( *p* = 0.08, OR = 2.812, 95% CI: 0.853–9.278) approached significance. In the multivariate analysis, NYHA classes III and IV ( *p* = 0.014, OR = 0.389, 95% CI: 0.183–0.826) independently predicted late mortality.

For patients with reoperative total arch surgery, survival at 8 years was 56 ± 12%.

**Fig. 2:**
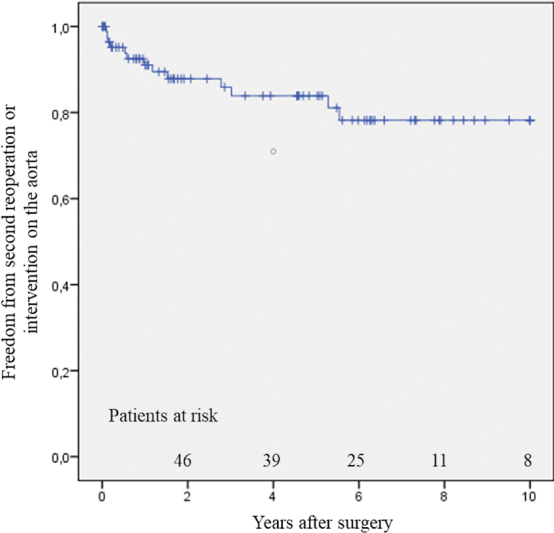
Freedom from second reoperation or intervention on the aorta.

During follow-up, 13 patients needed reoperation or reintervention, resulting in a linearized rate of 3.5%/year. The indications for second reoperation or intervention were chronic degenerative aneurysm in eight patients, chronic postdissection aneurysm in three, and contained rupture in two. These patients underwent an intervention or a second reoperation on the aorta, which included either thoracic or abdominal endovascular aortic repair in four and two patients, respectively, or combination of both interventions in another two patients. Debranching of the abdominal aorta was performed in two patients as an isolated procedure, or in combination with endovascular aortic repair in one patient. Open replacement of the entire thoracoabdominal aorta was performed in one patient. Second reoperation on the proximal aorta was performed in one patient with aortic root aneurysm. Freedom form reoperation at 1, 4, and 8 years was 91 ± 3%, 84 ± 5%, and 78 ± 6%, respectively ( Fig. 2 ). Using Cox's regression analysis of risk factors for second reoperation or reintervention on the aorta, the presence of chronic degenerative aneurysm approached significance ( *p* = 0.083, OR = 3.083, 95% CI: 0.787–12.079). Surgeon, aortic referral center, and extent of surgery were not predictive for late aortic events.

**Fig. 3:**
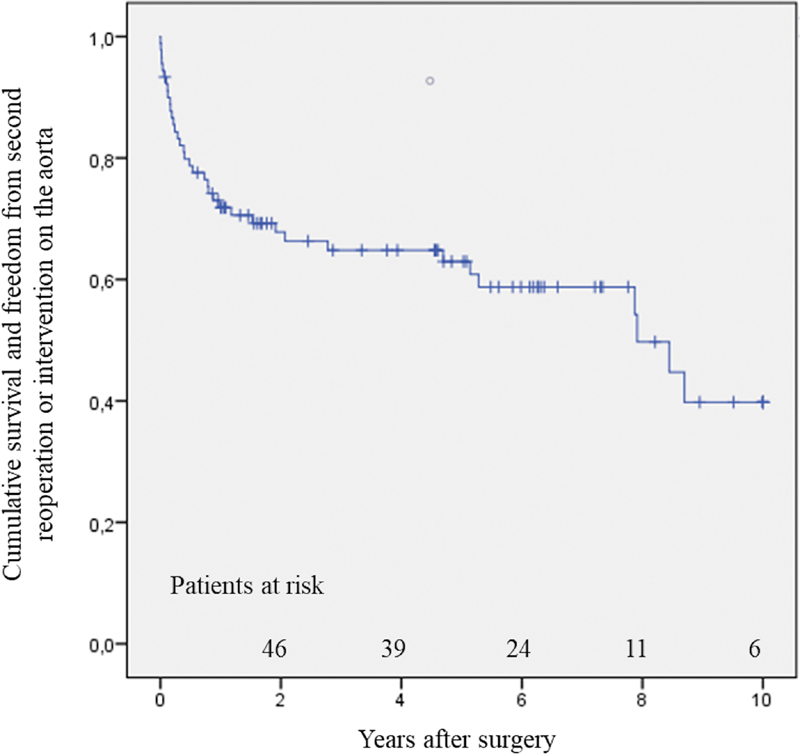
Cumulative reoperation- and intervention-free survival.

Cumulative reoperation- and reintervention-free survival for the whole group was 72 ± 5% at 1 year, 65 ± 5% at 4 years, and 50 ± 8% at 8 years ( Fig. 3 ).

## Discussion

In this study, we reviewed our two-center experience with reoperative aortic arch surgery in 90 patients after a previous operation on the proximal aorta. A standardized concept of mild systemic hypothermia has been used systematically, and, with exception of very few cases at the beginning of our experience, antegrade cerebral perfusion through at least one supra-aortic artery have been also routinely used. We found that, in patients undergoing reoperative aortic arch surgery, mild systemic hypothermia is safe and produces well-comparable outcomes with reports in deep hypothermia. 1 2 3 4 5 6 7 8 Furthermore, advanced age predicted operative mortality, and congestive heart failure with advanced functional impairment predicted late mortality. We also found that although the reoperation can cure the aortic arch disease, close follow-up of these patients is mandatory, as many of them develop considerable pathology in the downstream aorta.

In approaching patients requiring RAAS, several technical aspects are of utmost importance. Reentry of the thorax is a critical part of the operation, as dense adhesions of the right ventricle, the aortic graft or the innominate vein to the posterior sternal table renders these vital structures vulnerable to an increased risk of injury. A detailed preoperative CT scan of the thorax and the aorta should not only help plan the reentry into the thorax 17 but also provides clues for extrathoracic cannulation for CPB prior to reentry, if required. In such a way, the surgeon may avoid catastrophic reentry-related injuries. In a small portion of patients with aortas ruptured and contained within the anterior mediastinum, it may be required to place them safely on CPB and cool them to the desired level of systemic hypothermia prior to the repeat sternotomy, using one of the supra-aortic arteries for uninterrupted CPB and antegrade cerebral perfusion. 18 Furthermore, a considerable portion of these patients coming to a reoperation have advanced distal aortic pathology, requiring repair of the distal aortic arch and a portion of the descending aorta. Several recent technological advances, like the introduction of hybrid stent graft prostheses for frozen elephant truck, a technology available in Europe since 2005, have made extensive distal aortic repair in a redo setting more reproducible.

Reoperative arch surgery is not common surgical practice. The reoperation presents a tremendous challenge for the surgical team. Very few papers on RAAS have been published, mainly from aortic centers of excellence using systemic hypothermia extending in ranges from 18 3 4 to 25°C. 5 8 Reported rates of operative mortality range between 5 4 and 12%. 7 The observed rate of early mortality in this report was 6.7%, well comparable to other observations. 1 2 3 4 5 6 7 8 Several risk factors for early mortality have been reported, including previous Type A dissection, 5 older age, 3 and advanced NYHA class. 3 6 Most of the publications reporting on RAAS found that indeed prolonged CPB time 3 6 7 8 independently predicts operative mortality. One of the advantages of using mild systemic hypothermia (28–29°C), as opposed to deep hypothermia, is shorter CPB time required for cooling, especially rewarming of the patient. 19 With the understanding that CPB time is a risk factor for operative mortality, Preventza et al 20 have recently moved to higher temperatures as part of their organ protection strategy. 6 In our patient population, prolonged CPB time did not reach statistical significance in the multivariate analysis for operative mortality. Advanced age remained the only independent predictor for this adverse event.

Perioperative stroke in RAAS remains a dreadful complication with reported range between 3.6% 7 and 16%. 5 The observed incidence of stroke in the current report is 1%. Due to the low incidence of this event, neither risk analysis nor identification of risk factors could not be performed. We strongly believe that uninterrupted antegrade cerebral perfusion remains the backbone of contemporary cerebral protection strategy during surgery on the aortic arch, primary, or repeat. In both centers in this study, this technique has been solely used by four senior surgeons. The target temperature of the perfusate during the period of antegrade cerebral perfusion was kept at the range of 28 to 29°C, allowing for full preservation of cerebral autoregulation. Other authors reported stroke rates under 3% for total arch repair using a very similar cerebral protection strategy and systemic temperature management. 19 In a contemporary analysis of 145 patients with primary elective and emergent total arch repair (average ACP time of 55 minutes), Leshnower and coworkers, 19 at Emory University, found that temperature in range from 25 to 27°C did not represent an adverse risk factor for mortality, PND, TND, and renal or respiratory failure. 19 In patients undergoing primary 20 or reoperative aortic arch repair, 9 at the Texas Heart Institute systemic temperature, ranging between 24 and 28°C was also found to be safe.

Aortic events requiring intervention or reoperation in follow-up were common in this patient population. Others have reported similar observations. 8 Both institutions contributing to this work follow the patients at intervals both radiographically and clinically. Operative reintervention is performed as required. Freedom from reoperation at 8 years was 78 ± 6%. All second reoperations or interventions were performed in the downstream aorta, with the exception of one patient. No patient required second aortic arch reoperation. In analysis of risk factors for second reoperation or reintervention on the aorta, the presence of chronic degenerative aneurysm approached significance ( *p* = 0.083). Di Bartolomeo and coworkers 8 from Bologna reported that chronic postdissection aortic aneurysm was the only predictor of aortic reintervention, further pinpointing the need for a close follow-up. Late survival was 59% at 8 years, comparable to other reports. 3 5 6 8 We found that advanced functional impairment (with congestive heart failure in NYHA classes III and IV) independently predicted late mortality. Others have come to a similar conclusion. 6

## Conclusion

Mild systemic hypothermia with antegrade cerebral perfusion is safe for RAAS and produces well-comparable outcomes to those using deep hypothermia. Advanced age predicts operative mortality and advanced congestive heart failure predicts late mortality. Close follow-up of these patients is mandatory as many of them develop considerable pathology in the downstream aorta.
